# Efficiency determination of J-PET: first plastic scintillators-based PET scanner

**DOI:** 10.1186/s40658-023-00546-7

**Published:** 2023-04-08

**Authors:** S. Sharma, J. Baran, N. Chug, C. Curceanu, E. Czerwiński, M. Dadgar, K. Dulski, K. Eliyan, A. Gajos, N. Gupta-Sharma, B. C. Hiesmayr, K. Kacprzak, Ł. Kapłon, K. Klimaszewski, P. Konieczka, G. Korcyl, T. Kozik, W. Krzemień, D. Kumar, Sz. Niedźwiecki, D. Panek, S. Parzych, E. Perez del Rio, L. Raczyński, Shivani Choudhary, R. Y. Shopa, M. Skurzok, E. Ł. Stępień, F. Tayefi, K. Tayefi, W. Wiślicki, P. Moskal

**Affiliations:** 1 Faculty of Physics, Astronomy and Applied Computer Science, Jagiellonian University, prof. Stanisława Łojasiewicza 11, 30-348 Cracow, Poland; 2 Total-Body Jagiellonian-PET Laboratory, Jagiellonian University, 30-348 Cracow, Poland; 3 INFN, Laboratori Nazionali di Frascati, 00044 Frascati, Italy; 4 Faculty of Physics, University of Vienna, 1090 Vienna, Austria; 5 Department of Complex Systems, National Centre for Nuclear Research, 05-400 Otwock-Świerk, Poland; 6 High Energy Physics Division, National Centre for Nuclear Research, 05-400 Otwock-Świerk, Poland; 7 Center for Theranostics, Jagiellonian University, 31-034 Cracow, Poland

**Keywords:** Positron emission tomography, Registration efficiency, Efficiency of J-PET scanner, Medical imaging

## Abstract

**Background:**

The Jagiellonian Positron Emission Tomograph is the 3-layer prototype of the first scanner based on plastic scintillators, consisting of 192 half-metre-long strips with readouts at both ends. Compared to crystal-based detectors, plastic scintillators are several times cheaper and could be considered as a more economical alternative to crystal scintillators in future PETs. JPET is also a first multi-photon PET prototype. For the development of multi-photon detection, with photon characterized by the continuous energy spectrum, it is important to estimate the efficiency of J-PET as a function of energy deposition. The aim of this work is to determine the registration efficiency of the J-PET tomograph as a function of energy deposition by incident photons and the intrinsic efficiency of the J-PET scanner in detecting photons of different incident energies. In this study, 3-hit events are investigated, where 2-hits are caused by 511 keV photons emitted in $e^+e^-$ annihilations, while the third hit is caused by one of the scattered photons. The scattered photon is used to accurately measure the scattering angle and thus the energy deposition. Two hits by a primary and a scattered photon are sufficient to calculate the scattering angle of a photon, while the third hit ensures the precise labeling of the 511 keV photons.

**Results:**

By comparing experimental and simulated energy distribution spectra, the registration efficiency of the J-PET scanner was determined in the energy deposition range of 70–270 keV, where it varies between 20 and 100$\%$. In addition, the intrinsic efficiency of the J-PET was also determined as a function of the energy of the incident photons.

**Conclusion:**

A method for determining registration efficiency as a function of energy deposition and intrinsic efficiency as a function of incident photon energy of the J-PET scanner was demonstrated. This study is crucial for evaluating the performance of the scanner based on plastic scintillators and its applications as a standard and multi-photon PET systems. The method may be also used in the calibration of Compton-cameras developed for the ion−beam therapy monitoring and simultaneous multi-radionuclide imaging in nuclear medicine.

## Background

Positron emission tomography (PET) has become the central technique for detecting metabolically active malignant lesions by quantitative imaging of molecules labelled with positron emitters in the human body [1]. During the development of PETs, several challenges had to be overcome in terms of geometry, detector technology, data processing, and fast and efficient image reconstruction methods. The main challenge was to select the detector material and evaluate its performance. An ideal detector should have high interaction probability (efficiency), good spatial resolution (accurate photon interaction position information), good energy resolution (to suppress the random and scattered background), excellent time resolution (for efficient application of the TOF-based image reconstruction algorithm), and last but not least, low-cost fabrication to reach a wide market together with time (and thus cost) efficient calibration, data processing, and data handling. In the search for the ideal detector material for PET applications, various materials have been investigated, starting with sodium iodide (NaI(Tl)), bismuth germanate (BGO), barium fluoride ($$\hbox {BaF}_2$$), cesium fluoride (CsF), lanthanum bromide ($$\hbox {LaBr}_3$$), lutetium oxyorthosilicate (LSO), and yttrium-doped lutetium oxyorthosilicate (LYSO). Certain constraints on the properties of these detectors were considered to compare the characteristics required for an ideal PET scanner, such as hygroscopicity, effective atomic number (EAN), high light yield, rise and decay times of signals, and time and spatial resolution. Nowadays, most commercial PET scanners are based on LSO or LYSO detectors, which to a good extent have the important properties of an ideal PET scanner [2]. With advances in detector technology, the idea of total-body imaging PET (2 m long axial field of view (AFOV)) was revisited by the efforts of various groups e.g., uExplorer [3] in the framework of EXPLORER consortium (http://explorer.ucdavis.edu), PennPET Explorer [4], J-PET [5, 6] etc. An enhanced AFOV offers several advantages, such as performing dynamic whole-body imaging in a single scan due to a larger solid angle, a significant gain in effective sensitivity, a shorter scan time, and an increase in signal-to-noise ratio [1, 7, 8]. Two commercially available total-body scanners uExplorer and Biograph Vision Quadra are already established in clinical practice [3, 4, 9–11]. The cost of such a PET/CT scanner is estimated to be around $10 million for a single device [12], which poses an economic challenge for worldwide deployment [1, 2, 13–15]. One of the main factors behind the rising costs are the use of the expensive LYSO crystals (in total 564,480 crystals), which form multiple rings to achieve an axial coverage of 2 ms [16]. The community is actively looking for alternative ways to reduce the cost of large AFOV using various methods, such as sparse populations, developing flat panel devices, and resumption of BGO [17–19]. In the last decade, polymers have been proposed by the J-PET group as detector materials [20], as an alternative to inorganic scintillators, enabling the construction of low-cost scanners [20, 21]. Plastic-based scintillators are significantly cheaper than the widely used LYSO crystals. In addition, the longer attenuation of plastic scintillators for optical photons allows the use of longer scintillators and thus a larger AFOV. It is expected that the cost of building a total-body scanner based on plastic scintillators can be at least 4−8 times cheaper than scanners based on inorganic scintillators, including all major factors such as scintillators, AFOLV, electronics for signal readout and data acquisition(DAQ). The obvious reason that plastic scintillators have not been considered for PET scanners is primarily their low efficiency in registering 511 keV photons,  resulting in low imaging sensitivity. The PET sensitivity is proportional to the square of the detector efficiency and is one of the most important factors in evaluating scanner performance. The sensitivity of a PET scanner is defined as the number of 511 keV photon pairs detected per second and per unit of source activity (cps/$$\mu$$Ci or cps/MBq). However, it has been shown that efficiency can be increased by using multiple concentric layers [22]. The use of multiple layers can not only incur costs in terms of more acquisition units but also increase the complexity of data processing. The advantage of using plastic scintillators, on the other hand, is their excellent time resolution, which can reach up to 100 ps [22, 23], making them particularly suitable for TOF measurements required for the implementation of advanced reconstruction methods for PET imaging [24]. Due to the predominant hydrocarbon composition of the plastic scintillator, the photons interact mainly via Compton scattering, making it difficult to reduce the scattering background, which is crucial for good image quality. However, it has been demonstrated that the scattering fraction can be reduced to about 35% (NEMA-NU-2 norms) by accepting only those events where the energy loss in the interaction of the 511 keV photons is higher than 200 keV [5, 21, 25], which is comparable with other state-of-art PETs (PennPET (32%), uExplorer (35.8%), Siemens Biograph TM (31–34%) [2]). So far, a tomograph consisting of 3 concentric cylinders of plastic scintillators with an axial length of 50 cm has been put into operation [26]. In this prototype, the time over threshold (TOT) is used as a measure of energy deposition instead of a direct charge measurement. The measured values of TOT are correlated with the corresponding energy depositions using the fitting function obtained in previous work [27]. In this way, events can be filtered based on the energy deposition of the incident particles. However, it remains an open question to estimate the efficiency of J-PET as a function of energy deposition. The relationship between energy deposition and efficiency depends on the geometry of the detector and the settings of the electronics. This is important in view of the development of multiphoton tomography and, in particular, positronium imaging, which is of general interest in molecular imaging. community [6]. In addition, crystal-based detectors are being developed that register not only primary but also secondary scattering. For example, in view of applying quantum entanglement correlations to suppress the scatter fraction [28–31]. Therefore, such a method will be very useful for determining the relationship between registration efficiency as a function of energy deposition. Moreover, the method may also be useful for calibrating Compton imaging systems being developed for monitoring ion beam therapies as well as for simultaneous imaging of multiple nuclides in nuclear medicine [32–35]. The main goal of this work is to determine the efficiency of the J-PET scanner as a function of energy deposition and incident photon energies. This information will be useful in evaluating the performance of the J-PET scanner based on plastic scintillators for both standard 2-photon PET and multiphoton PET and positronium imaging [6, 36–38].

## Methods

### J-PET scanner

**Fig. 1:**
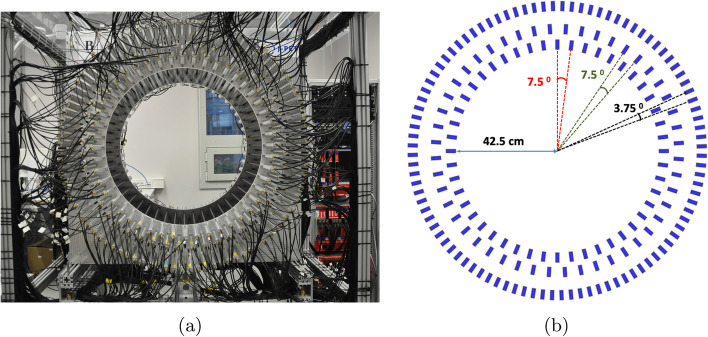
**a** Front view of the 192 modules prototype of J-PET scanner (see text for description). **b** Shows the cross section of J-PET with azimuth angular displacement of the scintillators arranged in 3 layers

Scanners based on plastic scintillators can be classified as TOF-PETs due to their excellent time resolution and fast decay time. The Jagiellonian Positron Emission Tomograph is the first prototype used for research purposes in a laboratory for the study of positron annihilation [6, 36, 39]. The J-PET group proposed using longer strips of plastic scintillators instead of the conventional small/pixel-sized crystal detectors to achieve a longer AFOV [20]. The use of the longer plastic scintillators is justified because of their lower light attenuation [40]. Signals are read from both ends of the scintillators. Therefore, a single detection module consists of a plastic scintillator (Eljen Technology EJ230) and two Hamamatsu R9800 vacuum tube photomultipliers (https://www.hamamatsu.com/us/en/index.html) connected to each end of the scintillator [21, 41]. The plastic scintillators are 50 cm long, 1.9 cm wide, and 0.7 cm thick [26]. A full-size prototype is shown in Fig. 1a. It consists of a total of 192 detection modules arranged in three cylindrical layers supported by aluminium plates. Figure 1b shows the angular displacement of the scintillation modules and the cross-section of the structure in a 2-D plane (front view). The first and second layers consist of 48 scintillators positioned at an azimuthal angular separation of 7.5$^0$, while the third layer consists of 96 scintillators arranged at an azimuthal angle of 3.75$^0$ to the centre of the scanner.

The diameter of the innermost layer is 85 cm, which limits the size of the objects to be scanned. The diameter of the second and third layers is 93.5 cm and 115 cm, respectively.

### Data acquisition

**Fig. 2:**
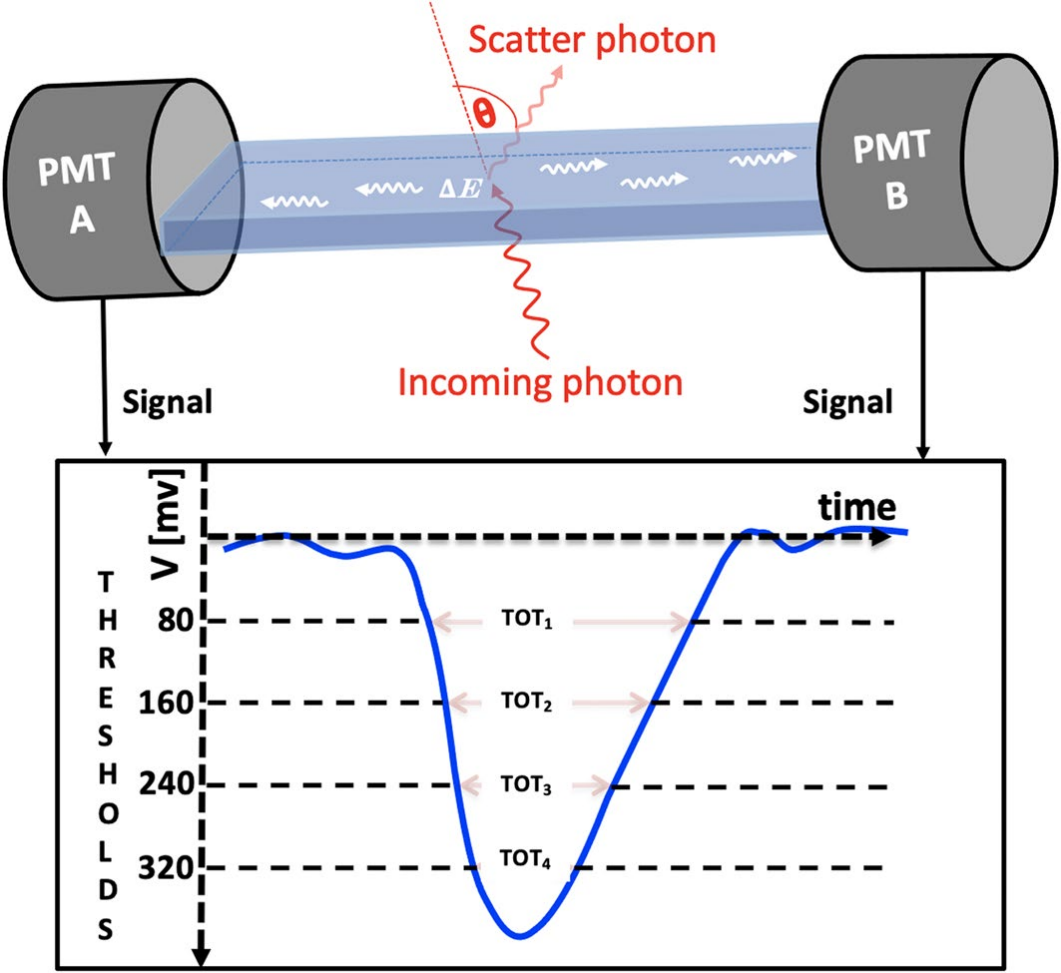
Illustration of deposition of energy by incident photons in the scintillator and obtaining the analog signal from both PMTs (A, B). The signals from each side are sampled at four equally spaced thresholds with minimum values of 80 mV and maximum values of 320 mV. The sum of $\hbox {TOT}_i$, where i is the number of thresholds, gives the measure of energy deposition calculated by Eq. 1

The incident particles, which deposit energy during the interaction inside the plastic scintillator, lead to signals from photomultipliers (PMT) attached to both ends of the scintillator (see Fig. 2). Both analog signals from the PMTs are sampled at four thresholds between 80 mV to 320 mV in the voltage domain (with an accuracy of 20 ps RMS) using multi-voltage-threshold (MVT) mezzaine [42] based on TRB3 boards [43, 44]. The signals from the MVT cards are sampled using TDCs implemented in the FPGA devices [45–47]. The signals from the plastic scintillators are very fast (rise time $\approx$ 0.5 ns, fall time $\approx$ 1.8 ns) [48] and tend to have much lower pileups compared to crystal-based detectors with an order of magnitude longer fall time [49]. It is estimated that even the longer plastic scintillators (upto 2 ms) can be used without pileups for PET imaging of patients receiving radio-pharmaceuticals with an activity of 500 MBq. To avoid additional dead time in the detection system, we use only the timing of the signal instead of direct charge measurement. This allows us to process higher data acquisition rates. The data are stored in a triggerless mode that can handle a data stream at rates of about 8 Gbps [50, 51].

**Fig. 3:**
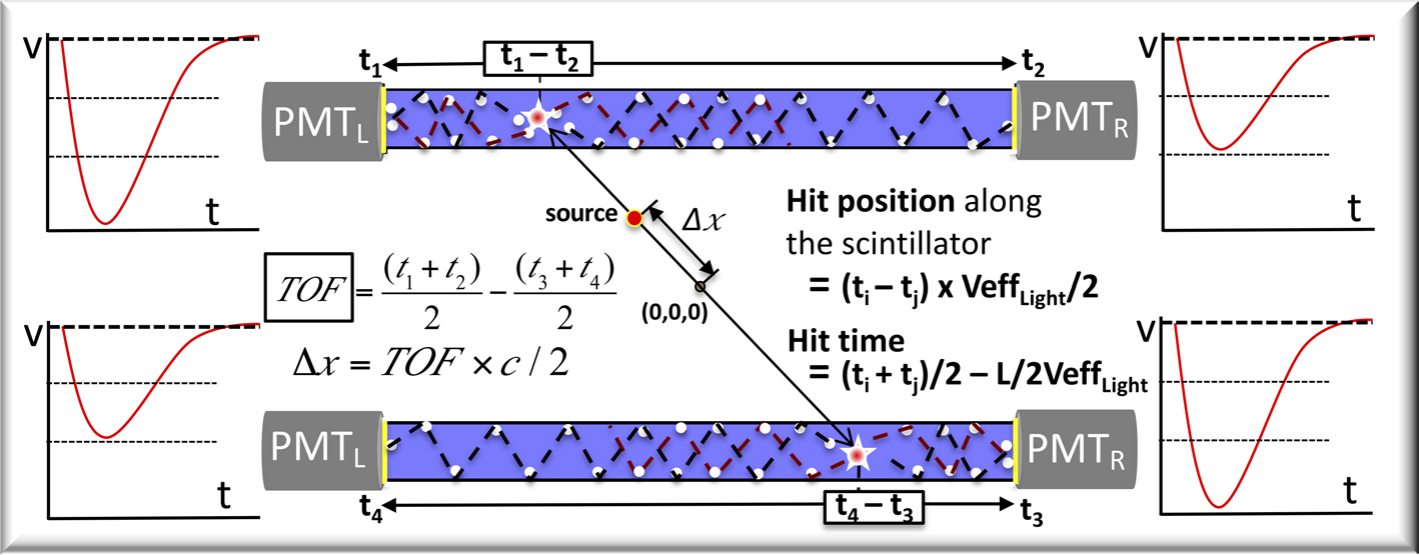
Demonstrates the principle of the J-PET scanner based on two plastic strips. Analog signals measured at four PMTs are also shown. For a scintillator of length L and considering the effective velocity of the light photons inside the scintillator ($\hbox {Veff}_\text{Light}$), the formula for calculating the hit position and hit time is shown in the figure. The annihilation point can be reconstructed from the measured TOF information between two hit positions (in different/opposite stripes)

Figure 3 explains the working principle used in J-PET to estimate the characteristics of photon interaction referred to as hit (hit time, hit position, etc.). The minimum criterion for successful registration of a hit is that the light signals arriving at both ends of the scintillator within a certain time coincidence window (6 ns) and the analog signals at both PMTs must have crossed the first threshold (80 mV). The hit time of the photon interaction in the scintillator is estimated from the measured average of the times of the light signals arriving at $\hbox {PMT}_L$ and $\hbox {PMT}_R$. Hit position, on the other hand, can be calculated by multiplying the measured difference of times at $\hbox {PMT}_L$ and $\hbox {PMT}_R$ by half the effective speed of light signal ($\hbox {Veff}_\text{Light}$) in the scintillator.

After calculating the hit positions of the registered photons produced by $\hbox {e}^+$ annihilation (511 keV), the line of response (LOR) can also be reconstructed. Thanks to the excellent time resolution of the plastic scintillators, the annihilation point along the LOR can be reconstructed with a good spatial resolution (Full Width at Half Maxima(FWHM) = 6.9 cm) by using the time-of-flight (TOF) information (with resolution 460 ps at FWHM for the coincidence resolving time (CRT) [6]). The TOT values summed over all four applied thresholds on the measured signals from both sides of the PMTs (L, R) give the estimate of the energy deposition by the interacting photons (see Fig. 2). The measured TOT is defined as:

1 $$\begin{aligned} \begin{aligned} \text{TOT} =&\, (\text{TOT}_{\text{th}_1}+\text{TOT}_{\text{th}_2}+\text{TOT}_{\text{th}_3}+\text{TOT}_{\text{th}_4})_{\text{PMT}_{L}} \\ {}&+\, (\text{TOT}_{\text{th}_1}+\text{TOT}_{\text{th}_2}+\text{TOT}_{\text{th}_3}+\text{TOT}_{\text{th}_4})_{\text{PMT}_{R}} \end{aligned} \end{aligned}$$

The relationship between energy deposition and TOT is not linear [27, 52–55]. In crystal-based PETs, selection of events based on energy deposition are used to reduce the scattering fraction. In the context of J-PET, the cuts can either be implemented based on the measured values of TOT or the energy deposition (can be determined from the values of TOT based on the relationship reported in the previous work [27]).

### Experimental setup and data measurements

**Fig. 4:**
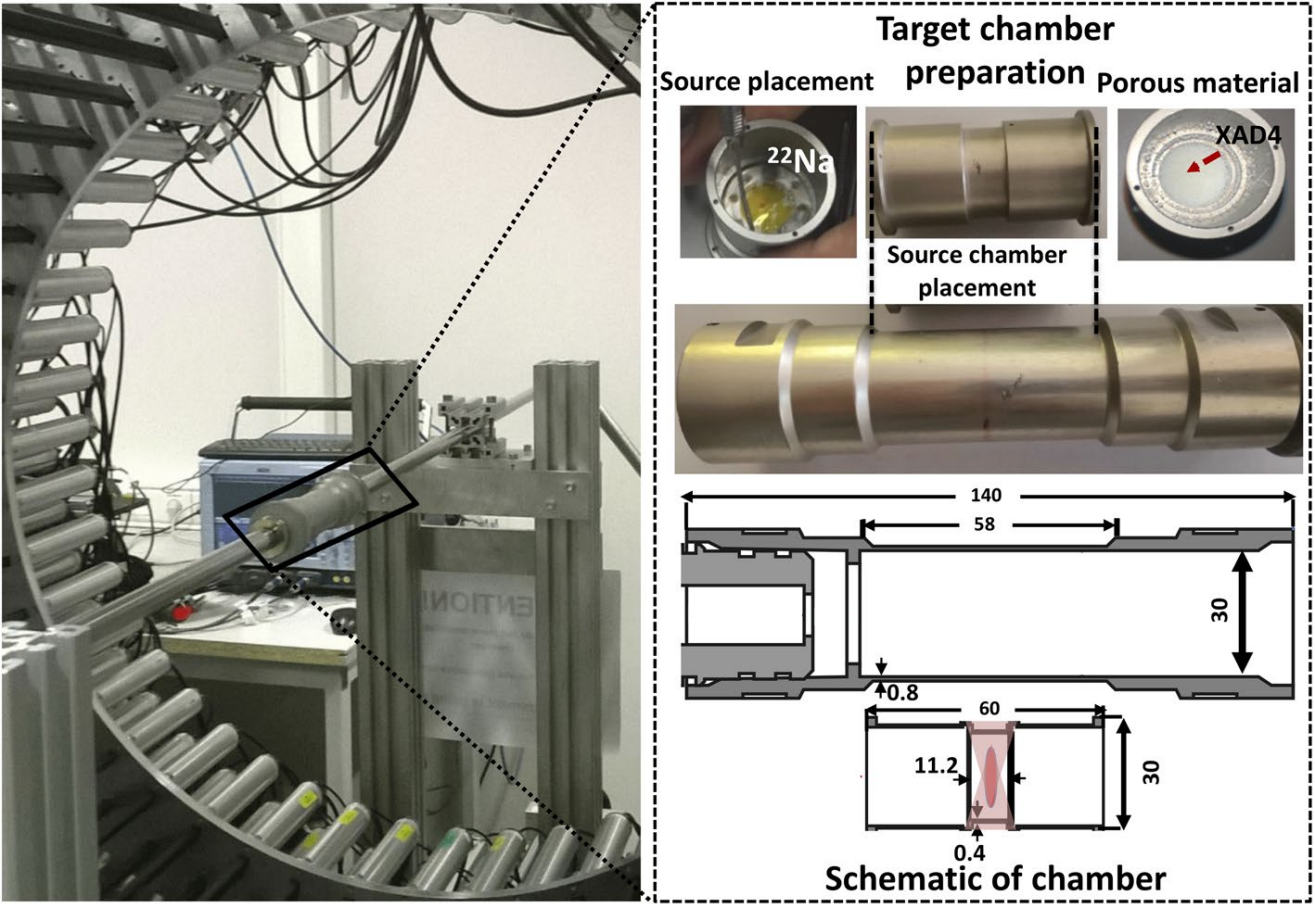
Left panel shows the experimental setup. The source chamber was aligned in the center of the J-PET tomograph. The upper part of the right panel shows the zoomed version of the source chamber and its preparation. The bottom part describes the schematic of the target chamber with dimensions (in mm). The red color in the center of the small cylinder represents the position of the source

For this experiment, a $^{22}$Na source ($\beta ^+$emitter) wrapped with a Kapton film was used. To place the source in the center of the scanner, a two-stage procedure was adapted (see Fig. 4). In the first stage, the source was placed in the center of a small cylinder made of a very thin aluminum layer, with the source surrounded by XAD-4 material (upper right corner of Fig. 4). In the second step, this small cylindrical chamber was inserted into a larger holder with a length of 14 cm and a diameter of 3.16 cm (in the center), so that the source was located in the center of the holder. Later, the holder was aligned with the center of the detector geometry, with the vertical support grounded outside the detector (see left inset in Fig. 4). The detailed specifications of the chamber were described in a previous work [56]. A $\beta ^+$ emitter source surrounded by porous material (XAD-4) increases the probability of positronium atoms (Ps) formation due to the interaction of emitted $\hbox {e}^+$ with the material [57]. The interaction of $e^+$ with the porous material leads to the emission of photons due to $e^+e^-$ annihilations. The photons escaping from the chamber can be attributed to two main origins.

First is due to the direct annihilation of $e^+$. The second is through the formation of Ps atoms, which can be formed mainly in two states, para-positronium (p-Ps) and ortho-positronium (o-Ps). In the case of p-Ps, the decay process is fast (within a few hundred picoseconds), while o-Ps decays with an average lifetime of 142 ns (in vacuum). The lifetime of o-Ps can be strongly affected by the porosity of the surrounding material, due to the possible contribution of pick-off annihilations (o-Ps$\rightarrow \hbox {2}\gamma$) [39]. In the XAD-4 material, for example, the average lifetime of Ps is 90 ns [57]. This allows the study of several interesting phenomena that directly correlate with the decay of Ps [58]. These phenomena include i.e., event-wise multiphoton registration, the average lifetime of Ps atoms, as well as fundamental physics studies of the angular correlations between annihilation photons emitted during the decay of Ps atoms and o-Ps decay Dalitz distributions [58]. In most cases, 2 back-to-back photons with energies of 511 keV are emitted, except for one channel (o-Ps) where 3 photons with a total energy of 1.022 MeV are produced, with the energy of the individual photons varying up to 511 keV [59]. J-PET scanner is based on the plastic scintillator (organic compounds with low atomic number). Photons interact mainly by Compton scattering with an effective cross section described by the Klein–Nishina formula [60, 61]. Therefore, an incident photon interacting with a plastic scintillator can deposit an energy directly related to its scattering angle. This means that photons with two different incident energies can deposit the same energies in the scintillator if they follow two different scattering angles. Therefore, it is difficult to identify the incident energy of the interacting photons based on the energy deposition. This is not the case with crystal-detector based tomographs where, a significant fraction of the photon interactions takes place via the photoelectric effect, especially at incident energies below a MeV range.

However, the energy of the incident photon can be conjectured based on various properties, which can be determined by studying the angular correlation of the registered photons by exploiting the J-PET geometry. To determine the efficiency of the J-PET scanner as a function of energy deposition and the energy of the incident photon, a detailed analysis was performed, which will be described in the next sections.

### Event selection

**Fig. 5:**
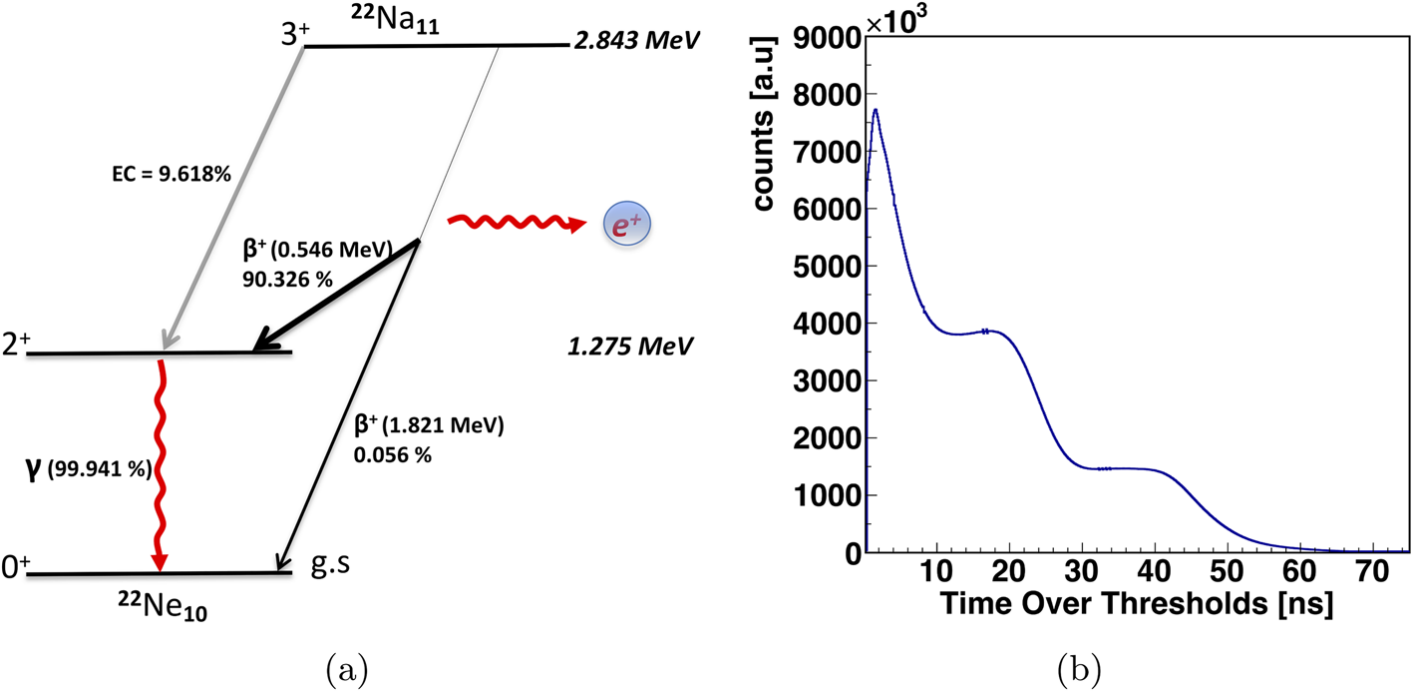
**a** Shows the decay scheme of the $^{22}$Na source. **b** shows the typical TOT spectrum for the $^{22}$Na source determined for events with 3 registered hits. The detailed description of the spectrum can be found in the text

In previous work [27], a relationship between TOT and energy deposition was established, allowing energy deposition to be directly pruned to reduce the amount of scatter. TOT values can be used as a preliminary criterion to distinguish the annihilation photons from the prompt gamma rays (1275 keV) also emitted by the $^{22}$Na source [62]. In the standard scanner, only the back-to-back annihilation photons (511 keV) are of interest. Therefore, to estimate the efficiency of J-PET from the scanner point of view, the registration of 511 keV photons is analyzed in the present work. Figure 5b presents a typical TOT spectrum for a $^{22}$Na source Fig. 5a, where 3 structures are clearly visible. The interacting photons deposit their energy mainly via Compton scattering in the plastic scintillator and therefore a continuous energy loss spectrum with the edges at 340 for 511 keV photons and at 1061 for 1275 keV gamma ray is expected. Since TOT is directly correlated with energy deposition, the Compton edge at a larger TOT value corresponds to the higher energy depositions. The Compton edge between 38 and 45 ns corresponds to the maximum energy deposition by prompt gamma rays (deexcitation gamma with energy of 1275 keV, see Fig. 5a), while in the middle it represents 511 keV photons (18–25 ns).

The first peak is a mixture of contributions from the scattering background when low energy is deposited and deposition by photons originating from the decays of o-Ps atoms into 3 photons, which can vary in their energies between 0 and 511 keV. Therefore, the values of TOT are used as preliminary selection criteria for event selection.

### Data analysis

**Fig. 6:**
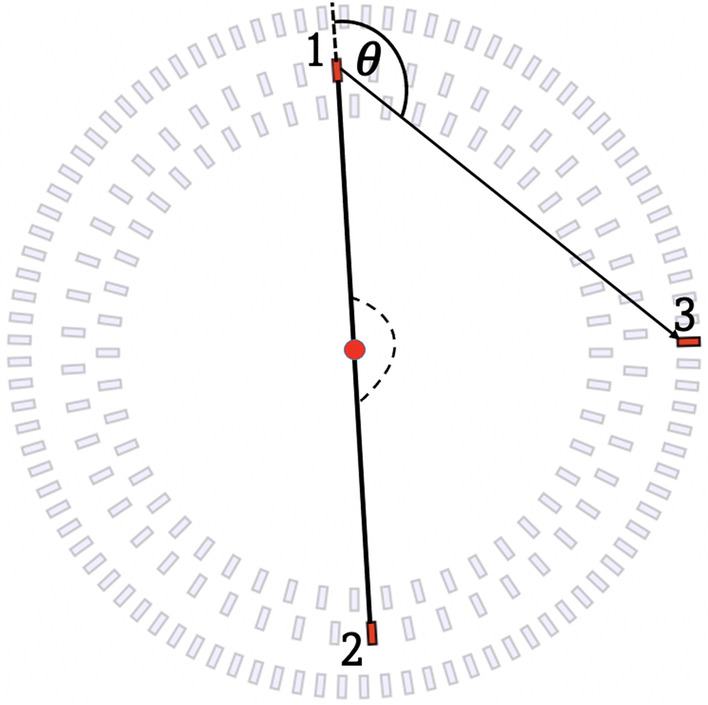
The definition of the analyzed events with 3 hits is displayed. Of the 3 hits, the first two hits (1,2) were caused by counter-propagating 511 keV photons. The third hit (3) represents the interaction by a scattered 511 keV photon. Here we see the association of the scattered photon (3) with the 511 keV photon marked 1, which gives us access to the scattering angle. However, the scattered photons are associated with the primary 511 keV photons based on the scatter test **S**, which can be described as the difference between measured times and calculated times (**S** = $\Delta \hbox {t}_{ij} - \hbox {D}_{ij}$/c), where ij represents the pair of primary and scattered photon, $\Delta$ $\hbox {t}_{ij}$ is the measured time difference, $\hbox {D}_{ij}$ is the distance between a pair of hits (31 or 32) and c is the speed of light

Events with 3 hits in a coincidence time window of 200 ns were analyzed. Of the total number of events, events with 3-hits account for only 1.2$\%$, but such strict criteria allows the analysis to be performed with a pure sample by filtering out the incorrectly selected hits of an event. In a 3-hit event of interest, two hits come from the 511 keV photons, and the third hit is caused by the subsequent scattered photon from one of these photons. Such an event is visually described in Fig. 6, where numbers 1 and 2 show the interaction of the primary back-to-back photons (511 keV), while 3 shows the interaction of the scattered photon. The aim is to measure the energy deposition by 511 keV photons as a function of the scattering angle ($\theta$) eventwise. With the correct identification of the primary photon and the measured scattering angle, the energy deposition can be calculated using Eq. 2 [63]:

2 $$\begin{aligned} \boxed {\Delta {E}=\textit{E}_{\text{inc}}\left[ 1-\frac{1}{1+\frac{\textit{E}_{\text{inc}}}{511~keV}\left( 1-\cos \theta \right) } \right] } \end{aligned}$$

where $\Delta {E}$ is the energy deposited by a photon of incident energy $\textit{E}_{\text{inc}}$ for a scattering angle $\theta$. Calculating the energy deposition of 511 keV photons by measuring their scattering angles requires the identification of photons with 511 keV energy. In addition to the preliminary criteria that depend on the measured values of TOT, a more stringent condition can be added based on the azimuthal angle difference of the registered photons.

**Fig. 7:**
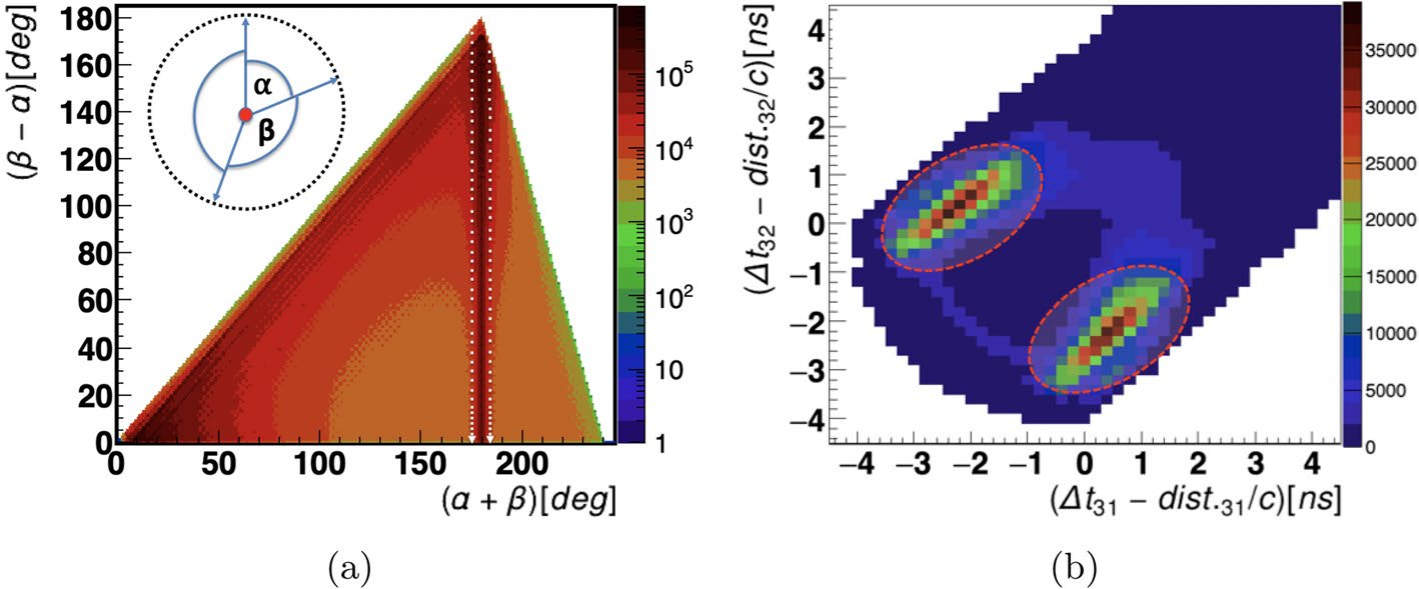
**a** The sum of the two smallest versus their differences angular correlations between the registered 3 hits are plotted event-wise. The number of events in a given pixel is shown in a logarithmic scale. The vertical white dashed lines indicate the region around 180 degree selected as events corresponding to two primary back to back 511 keV photons ($\alpha +\beta$ = 180$^0$) and one scatter photon. **b** Shows the results of the calculated scatter test. The elliptical dashed red lines show the regions used to assign the scattered photon (3 in Fig. 6) to the primary photon. The maximum centered around coordinate $(-2,0)$ indicates the events when photon 2 was scattered, while the maximum centered around $(0,-2)$ corresponds to the events in which photon 1 was scattered

It is known that for $e^+e^-$ annihilation in 2 photons (511 keV each), the photons are emitted in the back to back directions due to the conservation of momentum. In the 3-hit event selected for the current analysis, two hits from primary 511 keV photons and one from a scattered photon are expected (see Fig. 6). The assignment of the scattered photon to its primary photon is a crucial criterion that must be considered in order to obtain accurate scattering angle information. To select the hits corresponding to 511 keV photons, the angular correlation (azimuthal angle) between the 3 hits was calculated. The sum of the two smallest angles compared to their difference is shown in Fig. 7a.

**Fig. 8:**
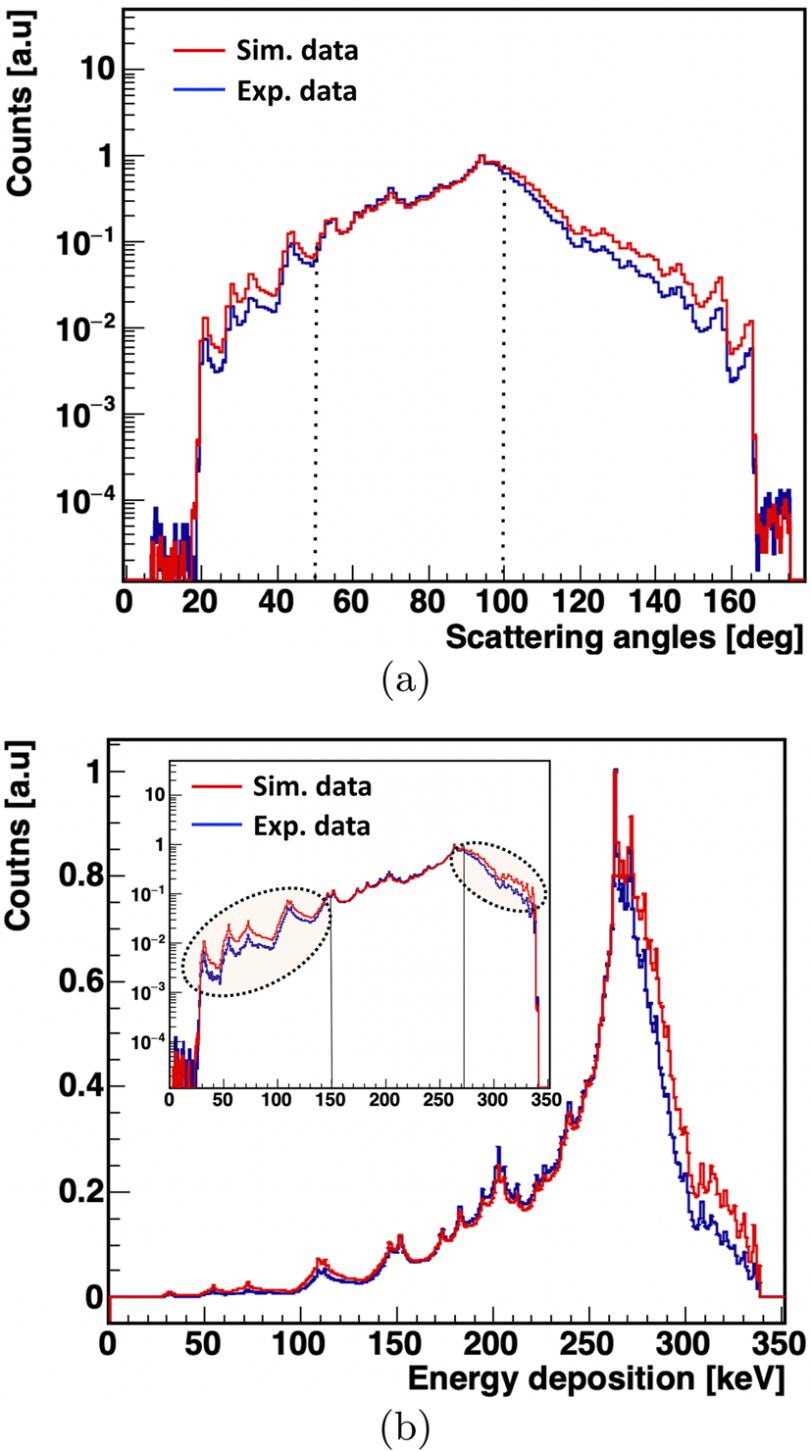
**a** Distribution of the scattering angles ($\theta$). **b** Distribution of the energy loss for tagged 511 keV photons. Results of the experiment and simulations are shown in blue and red, respectively. In the inset, energy deposition spectra are shown in a logarithmic scale

One can clearly see a band around 180 degrees (region between white dashed lines) representing the events where the sum of the smallest angles between two hits is 180$^0$, confirming that two hits are back to back (511 keV). The scatter test (**S**) was applied to verify the assignment of the scattered hit to the primary. The **S** test is based on the measured hit position (spatial coordinates) and hit time. After labelling the hits by 511 keV (1,2) and scattered photons (3), as shown in Fig. 6, **S** test was used to check whether 3 is associated with 1 or 2. The **S** test is calculated as the measured hit time difference of the test pair ($\Delta {t}_{31}$ or $\Delta {t}_{32}$) subtracted from the measured distance between hits ($D_{31}$ or $D_{32}$) divided by the speed of light (29.98 cm/nsec). Ideally, the **S** test should yield a value of 0 for the exact association. The study was event-driven. Figure 7b shows the results of the scatter test for all events analyzed. There is a clearly visible prolongation of the values of the **S** test close to 0, which could be due to the resolution of the hit time ($\approx$155 ps [26]) and hit position (4-5 mm for xy coordinates and 25 mm [64] for *z*). Only those scattering angles for which the value of **S**-test gave an approximation to 0 were selected, represented by the dotted elliptical shape (in Fig. 7b). The *x*-axis represents the value of **S** assuming that scattered photon (3) originates from photon 1, while the *y*-axis shows value of **S** for the hypothesis that photon 2 scattered. After assignment of hits to primary 511 keV and scattered photons the value of scattering angle $\theta$ was calculated for each event. Figure 8a presents experimental distribution of the scattering angle ($\theta$), and Fig. 8b shows the distribution of the energy deposition calculated using Eq. 2 from the known energy of the photon (511 keV) and its scattering angle ($\theta$). The structures observed in the figure are due to the geometrical configuration of the scintillator strips. The data were analyzed offline using a dedicated data analysis framework [65].

The registration efficiency of J-PET is determined as the ratio between the experimentally calculated energy deposition spectra and the simulated energy deposition distributions (“Registration efficiency of J-PET scanner” section). To obtain the simulated energy deposition spectra, Monte Carlo simulations were performed using the dedicated JPET-Geant4 package based on the Geant4 toolkit [66] (version 4.10.p02). JPET-Geant4 was developed with advanced features aimed at studying Ps decays from the perspective of the J-PET scanner [67]. To simulate the physics processes, we used the EmLivermorePolarizedPhysics model, which is commonly used to describe the interactions of electrons and photons with the matter in the energy range from 10 eV to 100 GeV using the Livermore library interpolated data tables. This model can be used to simulate the following physics processes: Photoelectric effect, Compton scattering, gamma conversion, Rayleigh scattering, ionization, and bremsstrahlung. For each event, three primary photons were generated, two of them 511 keV photons (in the back-to-back direction) and one prompt photon (1274.6 keV) emitted isotropically with respect to the direction of 511 keV photons. The simulated data were analyzed using the J-PET data analysis framework [65]. For a realistic simulation, the interaction of photons in the aluminium target chamber was also taken into account. The generated photons were registered in a 3-layer J-PET geometry consisting of plastic scintillators wrapped with Kapton foils. Hit time and hit positions were smeared with a time resolution ($\sigma _t$) of 155 ps and a spatial resolution of 25 mm ($\sigma _z$), respectively. Events with 3 hits were analyzed according to the same selection criteria as used for the experimental data, except that the TOT cut was replaced by the maximum energy deposition of 511 keV. To mimic the minimum energy deposition required to register a hit, alike in experiment, the energy deposition values were first smeared with a resolution of $\sigma \left( E \right) /E=0.44/\sqrt{E\left[ MeV \right] }$ (as reported by Moskal et.al. [21]) and later a threshold value for the minimum energy deposition was set to 70 keV, as predicted by the fitting function in a previous work [27]. The simulations were validated by generating control spectra that were used for the experimental data. The first control spectrum was the sum of the two smallest angles against the difference of their angular correlation and the second control spectrum was the scattering test distribution. For comparison with the experimental data, the energy deposition spectrum was calculated based on the estimated scattering angles according to the assignment of the scattered hits based on the **S** test. Figure 8a shows the comparison of the experimental (blue line) and simulated (red line) values of the scattering angles.

There are a few points worth discussing here. In general, the structure in Fig. 8a reflects the geometrical configuration of the scanner and the angular distribution of the scattered photons. In particular, the highest probability for the secondary interaction is when the photon is scattered at about 90 degrees as can be inferred from Fig. 8a. It is clear that events where 511 keV photons are scattered at smaller angles ($20^0$), corresponding to lower energy deposition, are suppressed in both the experimental and simulated scattering angle spectra, which could be due to a high first threshold (80 mV in the experiment) that is mandatory for a hit to be registered. The range between 20$^0$–95$^0$ shows the difference between the experimentally obtained and the simulated scattering angle distribution. At scattering angles larger than 100$^0$ there is a sharp drop in the measured scattering angles. This can be explained by the fact that at higher angles the scattered photon has a lower chance to interact with another scintillator strip. The scattering angles calculated for each event are converted to energy deposition using Eq. 2, as shown in Fig. 8b. In this figure (inset), the logarithmic scale is shown to illustrate the (non)agreement between experimental and simulated energy deposition spectra. In the scientific literature, the efficiency of a detector is generally described as a function of the energy of the incident photon, either as absolute efficiency: ratio of registered signals to the number of photons emitted by the source, or as intrinsic efficiency: number of registered signals relative to the number of photons interacting in the detector as a function of the energy of the incident photons. It is also important to estimate the efficiency as a function of energy deposition to accurately understand the response of plastic scintillators, especially at longer axial lengths. There is limited information in the literature on calculating efficiency as a function of energy deposition [68]. In the present work, we first estimated the registration efficiency as a function of energy deposition, based on which the intrinsic efficiency of the J-PET scanner was calculated as a function of the energy of the incident photon. The results are presented in the next section.

## Results

### Registration efficiency of J-PET scanner

Registration efficiency was estimated by dividing the energy deposition spectra obtained from the experimental data by the simulated data. It is worth noting that in both cases the energy deposition was calculated by measuring the scattering angles of 511 keV photons event by event. Finally, the experimental energy deposition spectra were divided by the simulated spectra. In Fig. 8b, the difference reflects the efficiency of the registration. In the case of simulated spectra, a 100$\%$ efficiency was assumed, therefore, the difference between the experimental and MC spectra reflects the experimental efficiency of photons registration.

**Fig. 9:**
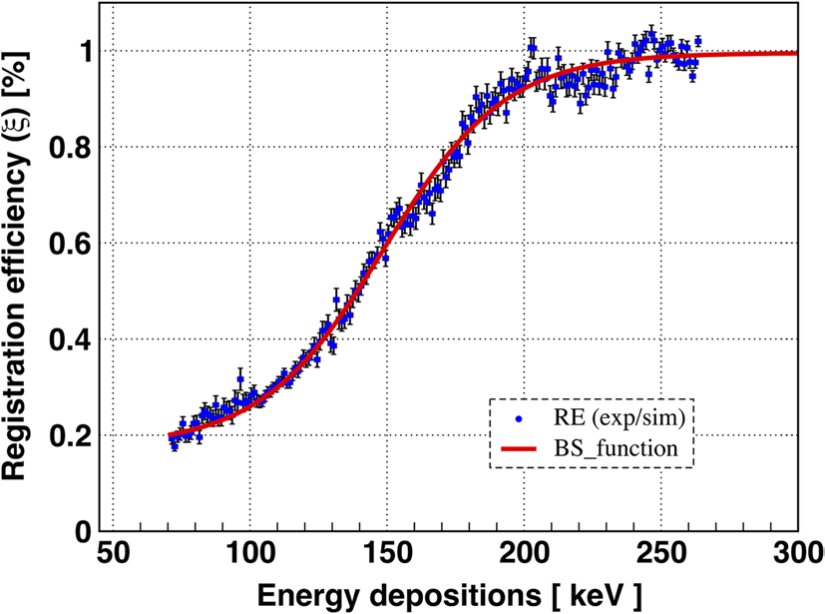
Registration efficiency is shown by blue symbols as the ratio of energy depositions spectra (Exp/Sim). The error bars indicate the statistical uncertainties. The energy range starts at 70 keV, since the simulations were performed with a threshold energy deposition of 70 keV. The red line shows the Boltzmann Sigmoid Function: BSF =$A_1+(A_0-A_1)/\left( 1+exp\left( \left( A_2-\textit{E}_\text{dep} \right) /A_3 \right) \right)$, where $\hbox {A}_0$ = 0.177 ± 0.005 and $\hbox {A}_1$= 0.996 ± 0.003 represent the minimum and maximum values of BSF, respectively. $\hbox {A}_2$ represents the mean between the minimum and maximum values of BSF, which is 148.7 ± 0.5 keV, while $\hbox {A}_3$ = 22.77 ± 0.48 keV indicates the slope of the function. The BSF is plotted with 3$\sigma$ standard deviations. $\sigma$ is calculated using the covariance matrices as a function of the uncertainties in the values of the parameters ($\hbox {A}_0, \hbox {A}_1, \hbox {A}_2, \hbox {A}_3$). $\hbox {\textit{E}}_\text{dep}$ is the value of the energy deposition. The goodness-of-fit parameter ($\chi ^{2}$/ndf) is equal to 334/189

It is expected that registration efficiency decreases with the decreasing energy deposition. Indeed for low values of deposited energies, the experimental spectrum is below the simulated one. However, such a tendency is also observed for the high energy losses where the expected experimental registration efficiency is equal to 100$\%$. This is because the events used for the analysis include signals from 511 keV as well as from the scattered photons. If the energy loss of the primary 511 keV photon is high then the energy of the scattered photon and hence the energy loss of the scattered photon is low resulting in lower detection efficiency of the full event.

Finally, the registration efficiency of the J-PET scanner as a function of energy deposition is calculated by dividing the experimentally obtained energy deposition spectra with the spectra predicted based on the Geant4 simulations. Before the division, both spectra are normalized to the unit value for the most probable energy deposition ($\approx 270$ keV, see Fig. 8b). The result is plotted (blue symbols with error bars) in Fig. 9. It can be interpreted as follows: a hit is registered with a maximum probability equal to 1, when the deposited energy is equal to or greater than 270 keV. In the cases when the deposited energy is smaller than this value, the probability of registration decreases. The obtained ratio is fitted with the Boltzmann sigmoid function. BSF is a modified version of the sigmoid function (correlating the sigmoid function with the Boltzman distribution) with extended parameters that can be used intuitively to describe the behavior of changes in registration efficiency as a function of energy loss between a range of minimum and maximum values. The fitted function with the parameters, their interpretation, and values are shown in the caption of Fig. 9.

### Intrinsic efficiency of J-PET scanner

**Fig. 10:**
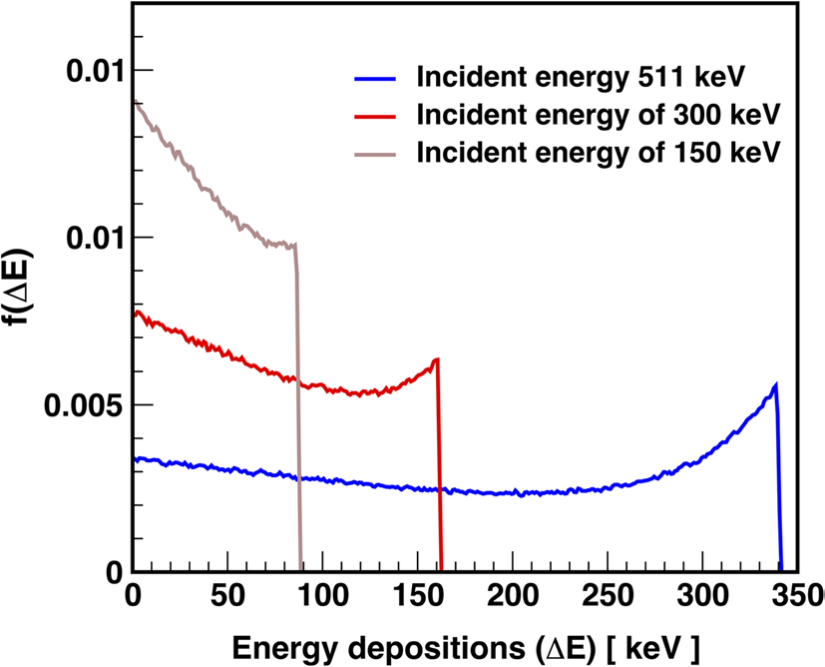
Normalized Klein–Nishina function describing the probability density distribution of energy depositions by photons of different incident energy in a plastic scintillator

To convert the registration efficiency ($\xi$), which is a function of energy deposition, into the intrinsic efficiency of the J-PET scanner as a function of the energy of the incident photon, the probability of a given energy deposition by the incident photon in the scintillator must be calculated. For this purpose, the Klein–Nishina function was used for the different energetic photons (between 150 and 511 keV). Three exemplary cases are shown in Fig. 10.

Later, the spectra were normalised so that the sum over the total energy depositions distribution is $\int _{\Delta _{E=0}}^{\Delta \textit{E}_\text{max}}f(\Delta E)\text{d}(\Delta E)=1$. Finally, the registration efficiency spectra were corrected and integrated over all deposited energies, yielding an estimate of the intrinsic efficiency of the J-PET scanner for the selected incident photon energy. The formula can be expressed as follows:

3 $$\begin{aligned} \boxed {\mathrm{J-PET}_\mathrm{Eff.}(\textit{E}_\text{inc}) = \int _{\Delta E=0}^{\Delta \textit{E}_\text{max}(\textit{E}_\text{inc})} f\left( \Delta E \right) *\xi \left( \Delta E \right) \text{d}(\Delta E)} \end{aligned}$$

The Klein–Nishina distribution normalized to unity gives the probability density distribution f($\Delta E$) for the energy depositions of an incident photon inside the plastic scintillators.

**Fig. 11:**
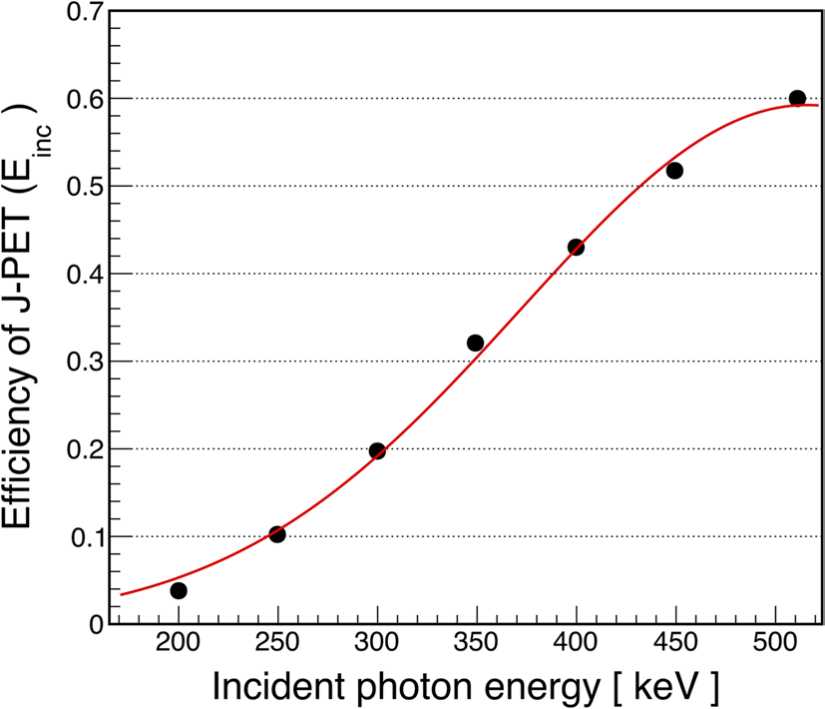
The intrinsic efficiency of the J-PET scanner as a function of the energy of the incident photon is shown by the black-filled circles. The red line shows the fitting function described in the text

The intrinsic efficiency of the J-PET scanner was calculated as a function of the energy of the incident photon (under the condition that the photon interacted in the plastic strips). It was calculated for different incident energies as shown by the black-filled circles in Fig. 11. The data points are fitted with the function (Bell shape function) = $p_{0} *\text{exp}(-(x-p_{1})^{2} / (2 *p_{2}^{2})$, where $p_{0}$ = 0.59 ± 0.02, $p_{1}$ = 515.80 ± 11.89 keV and $p_{2}$ = 143.80 ± 7.86 keV, respectively.

## Discussion

The registration efficiency of the J-PET scanner was estimated as a function of energy deposition. To this end, an algorithm was developed to identify the 511 keV photons and measure their scattering angles, which eventually allowed the energy deposition to be calculated. For this purpose, events with recorded 3-hits were analyzed. The coincidence time window (CTW) for the hits in the events was set to 200 ns. However, after applying the selection criteria to the primary and the scattered photon, events with hits recorded within a CTW of $\approx$ 4 ns remained for further analysis. To estimate the efficiency of registration, the $\hbox {\textit{E}}_\text{dep}$(exp) distribution was divided by the corresponding simulated distribution ($\hbox {\textit{E}}_\text{dep}$(sim)). In the simulations, each hit is registered under the only condition that the energy deposition in the interacting photons is above the specified threshold (70 keV). The readout electronics is therefore neglected. In the measured data, on the other hand, hits are registered when the amplitude of the signals generated at both ends of the scintillator is larger than the value of the first fixed threshold. This can lead to suppression of hits with lower energy deposition (or scattering angles), especially for hit positions near the edges of the scintillators, which is not the case for simulated hits. The registration efficiency function was extracted by fitting the ratio of $\hbox {\textit{E}}_\text{dep}$(Exp) and $\hbox {\textit{E}}_\text{dep}$(Sim). From the function (see Fig. 9), it can be concluded that the probability of hit registration increases with energy deposition, reaching the value of unity (100$\%$) at the energy loss of 270 keV. The registration efficiency was estimated as a function of energy deposition, which was later converted to an intrinsic efficiency as a function of incident photon energy. The estimate of the intrinsic efficiency is derived from the efficiency of the detector as a function of the energy of the incident photon, including signal readout effects. This study has certain limitations in optimizing the method presented. The first limitation is the labeling of the true annihilation and scattering candidates. The algorithm developed to label the 511 keV photons selects the annihilation candidates well based on their angular correlation and their reconstructed annihilation points (which should be within the radius of the chamber used). Considering the diameter of the chamber (3.16 cm) and the angular difference window used (4$^0$), the possibility of incorrect selection of annihilation candidates cannot be completely neglected. Another limitation is to assign the interaction of the scattered photons to their primary origin in order to calculate the scattering angle, and for this purpose, the scattering test (**S**) was proposed. The scattering test relies on two important properties associated with registered interactions of photons, namely spatial and time resolution. For the present work, it is of great importance to apply the correct resolutions for each interaction in order to compare the simulated results with the experimental ones. The angular resolution depends on the distance between the interactions of primary and scattered photons. The larger the distance between interactions, the better is angular resolution. The uncertainty in the positioning of the hit ($\sigma$ = 2.5 cm in *z*) affects the angular resolution, which varies between 7$^0$ (for the shortest distance $\approx$ 12 cm) and 1.5$^0$ (for the longer distance $\approx$ 100 cm) for the FWHM. In the case of the 511 keV photon, such uncertainty in the *z* position may smear the calculation of the energy deposition of $\approx$ 12 keV and $\approx$ 1 keV, respectively. These values may vary slightly from scintillator to scintillator. The values chosen here are based on the characteristic studies performed earlier. For different values of the resolutions, the scattering angle distribution may vary, but not significantly. It is also found that the scattering test predicts the correct assignment of the scattered photon in about 98$\%$ of cases, provided the annihilation photon is correctly labeled. Changing the resolution may decrease/increase the number of events of interest, but not the shape of the distribution, and therefore should not change the final conclusion of the present study. It should be added that for successful implementation of this method for the registration of photons with lower incident energy, it is necessary to optimize the lowest threshold value, which is the criterion for qualifying the signals to be registered, especially for registration of the scattered photons, which have even relatively low energies. Thus, if you set a higher value for the lowest threshold, the registration of the scattered photons and thus of the whole event will be rejected. Furthermore, the criteria to register the signals at both ends of scintillators may also suppress some good events to be registered.

## Conclusion

In this article, we present a method for estimating the registration efficiency of detectors based on plastic scintillators in which the primary mode of photon interaction is Compton scattering. The potential of the method is demonstrated using data measured with the J-PET detector, which consists of 192 plastic scintillators. The registration efficiency takes into account the effects of the signal readout chain after the interaction of photons in the detector. For the first time, the registration efficiency of J-PET is estimated as a function of the energy deposited by the photons and later converted to the intrinsic efficiency of J-PET as a function of the energy of the incident photons. The determined registration efficiency as a function of the deposited energy and the intrinsic efficiency as a function of the energy of the incident photons are crucial for improving the quality of the standard PET images and of particular importance for the corrections of multiphoton images (e.g., due to the formation of Ps atoms, $e^+e^-$ $\rightarrow$ 3$\gamma$), where the energy of the registered photons varies between 0 and 511 keV [36, 37]. Moreover, for positronium decay-based studies in J-PET, such as positronium imaging [6, 13, 69] and the test for discrete symmetries [36, 58, 70], it is necessary to suppress background scattering [71, 72]. In the presented measurement, the lowest threshold of 80 mV suppressed the hits with low energy depositions. Therefore, the obtained function for registration efficiency applies to values above 70 keV (Fig. 9). It is planned to lower and optimize the threshold in the next measurements to determine the registration efficiency function for deposits with the lowest possible energy, which is especially important for positronium imaging studies with the J-PET scanner.

## Data Availability

The data that support the findings of this study are available from the corresponding author upon request.

## Abbreviations

PET: Positron emission tomography
NaI(Tl): Thallium--doped sodium iodide
ECAT: Emission computed axial tomograph
EAN: Effective atomic number
BGO: Bismuth germanate
LSO: Lutetium oxyorthosilicate
$$\hbox {BAF}_2$$: Barium fluoride
CsF: Cesium fluoride
$$\hbox {LaBr}_3$$: Lanthanum bromide
LYSO: Lutetium-yttrium oxyorthosilicate
AFOV: Axial field of view
TOT: Time over thresholds
PMT: Photomultipliers
RMS: Root-mean-square
MVT: Multi-voltage-threshold
$\hbox {Veff}_\text{Light}$: Effective speed of light signal
LOR: Line of response
TOF: Time of flight
Ps: Positronium atoms
p-Ps: Para-positronium
o-Ps: Ortho-positronium
$$^{22}$$Na: Sodium-22
RE: Registration efficiency
BSF: Boltzmann sigmoid function
